# Construction and validation of a nomogram for detecting chronic kidney disease in patients with nonalcoholic fatty liver disease: Insights from the NHANES database

**DOI:** 10.1016/j.clinsp.2025.100686

**Published:** 2025-05-07

**Authors:** Dazhang Deng, Yutong Xie, Ya Wang, Wanhan Song, Yuguo Liu, Bin Liu, Honghui Guo

**Affiliations:** a Department of Nutrition, School of Public Health, Guangdong Medical University, Dongguan, PR China; b Laboratory of Hepatobiliary Surgery, the Affiliated Hospital of Guangdong Medical University, Guangdong Medical University, Zhanjiang, PR China; c Dongguan Key Laboratory for Development and Application of Experimental Animal Resources in Biomedical Industry, School of Public Health, Guangdong Medical University, Dongguan, PR China

**Keywords:** Chronic kidney disease, Nonalcoholic fatty liver disease, Nomogram, Prediction Model, NHANES

## Abstract

**Background and objectives:**

Fatty liver disease is often associated with renal impairment in many patients. Early detection and prompt intervention are crucial for improving patient quality of life and reducing mortality rates. This study aimed to develop and validate a nomogram for detecting the risk of Chronic Kidney Disease (CKD) comorbidity in adults with Nonalcoholic Fatty Liver Disease (NAFLD) in the United States.

**Methods:**

From the NHANES (2017‒2020) database, the authors enrolled 2848 NAFLD participants, of whom 633 also had CKD. The authors employed the Least Absolute Shrinkage and Selection Operator (LASSO) regression and multivariate logistic regression to identify variables with predictive value. The overlapping features were selected to construct a predictive model, which was presented as a nomogram. The effectiveness of the nomogram was evaluated using Receiver Operating Characteristic (ROC) curves, calibration plots, and decision curve analysis.

**Results:**

Six indicators were included in the model: age, systolic blood pressure, serum albumin, high-sensitivity C-reactive protein, total cholesterol, and triglycerides. The area under the curve of the nomogram for predicting CKD in the training set was 0.772, with a 95 % Confidence Interval (95 % CI) of 0.746 to 0.797. In the validation set, the area under the curve was 0.722, with a 95 % CI of 0.680 to 0.763. The calibration curve analyses demonstrated that the prediction outcomes of the model aligned well with the actual outcomes, indicating good clinical applicability.

**Conclusions:**

The nomogram demonstrated excellent performance and has the potential to serve as an auxiliary tool for detecting CKD in NAFLD patients.

## Introduction

Non-Alcoholic Fatty Liver Disease (NAFLD), now referred to as Metabolic Dysfunction-Associated Steatotic Liver Disease (MASLD), has increasingly become one of the most prevalent chronic liver disorders worldwide. Current estimates indicate that the global prevalence rate for NAFLD may reach up to 24 %, affecting approximately 80 to 100 million individuals in the United States alone.^1^^,^^2^ NAFLD begins with the accumulation of ectopic fat in the liver, which is further exacerbated by increased fatty acid production resulting from excessive intake of nutrients, primarily glucose, fructose, and fatty acids.^3^ This accumulation is followed by subsequent impairment of the liver's stress response. Despite the incomplete understanding of the pathogenesis of NAFLD and potential interindividual variations, a growing body of evidence suggests that NAFLD is a multisystem disorder with implications for extrahepatic organs and regulatory pathways.^4^ The clinical burden of NAFLD extends beyond hepatic morbidity and mortality; for instance, the presence of NAFLD significantly increases susceptibility to type 2 diabetes mellitus, cardiovascular disease, and Chronic Kidney Disease (CKD) .^5^ Furthermore, studies have shown that patients with hepatic disorders are at risk for both acute and chronic renal impairment.^6^

Numerous observational studies have provided compelling evidence supporting the correlation between NAFLD and CKD, as well as their coexistence in clinical outcomes.^7^^,^^8^ CKD is characterized by structural or functional abnormalities in the kidneys, which are assessed using a comprehensive set of variables, including glomerular filtration rate, albuminuria threshold, and the duration of renal injury.^9^^,^^10^ Due to the insidious nature of chronic kidney injury, it is often detected only in end-stage patients. For example, individuals with liver cirrhosis are susceptible to hepatorenal syndrome and may require combined liver-kidney transplantation,^11^ indicating that concurrent liver and kidney injury may occur in the early stages. It is crucial for individuals with CKD to have a thorough understanding of their condition, as lifestyle modifications can significantly influence the progression of CKD.^12^ Mao et al. found that dietary patterns affect kidney function, with a low animal and high plant diet being beneficial for renal health.^13^ This suggests that dietary interventions may serve as an effective strategy to mitigate the decline in kidney function. Additionally, Hu et al. also demonstrated that short-term weight loss, along with the maintenance of a normal waist circumference and waist-to-hip ratio, positively impacted the progression of CKD in patients with NAFLD.^14^ Therefore, early detection and timely intervention for CKD in patients with NAFLD can effectively slow disease progression.

With the rapid advancement of machine learning, an increasing number of statistical prediction models have become widely utilized across various domains of clinical research. Among these models, nomograms offer distinctive advantages due to their ability to visually represent the outcomes of logistic regression, thereby facilitating the straightforward calculation of disease risk.^15^^,^^16^ The growing adoption of this method by medical researchers for predicting the occurrence and progression of various diseases can be attributed to its robustness, reliability, and significant utility. Consequently, nomograms can serve as a valuable tool for predicting the likelihood of CKD, and the calculated results can provide a foundation for clinicians to implement early interventions in patients. For instance, Chowdhury et al. developed a predictive nomogram for CKD in patients with type 1 diabetes mellitus using readily available routine examination data to detect early stages of CKD.^17^

Previous studies have primarily focused on single diseases such as NAFLD or CKD, there has been limited research on comorbidities. Numerous studies have demonstrated a significant association between NAFLD and CKD. Given that CKD exhibits discernible manifestations only in its advanced stages, these two conditions may coexist in the early phases of the disease. Therefore, the objective of this study was to develop and validate a risk prediction model utilizing routine clinical examination indicators for NAFLD based on the National Health and Nutrition Examination Survey (NHANES) database. This model aims to facilitate the timely detection of CKD in adults with NAFLD and presents the findings visually through a nomogram.

## Methods

### Study design and participants

The present study employed a cross-sectional design, utilizing data extracted from the NHANES database. The Liver Ultrasound Transient Elastography data from NHANES (2017‒2020) included a total of 10,409 samples, of which 5076 samples were identified as having hepatic steatosis based on the screening criteria of a Controlled Attenuation Parameter (CAP) ≥238 dB/m, while excluding those with missing data.^18^ In alignment with the study's objective and the definition of NAFLD, the exclusion criteria were as follows: 1) Excessive alcohol consumption; 2) Positive for Hepatitis B surface Antigen (HBsAg) and/or Hepatitis C RNA; 3) Age less than 18-years; and 4) Missing data. Excessive alcohol consumption was defined by the Dietary Guidelines for Americans (DGA) as more than 2 drinks per day for men and more than 1 drink per day for women. Consequently, a total of 2848 participants with NAFLD were selected from the database. The Glomerular Filtration Rate (GFR) for each NAFLD participant was calculated in accordance with the clinical practice guidelines of Kidney Disease Improving Global Outcomes (KDIGO), utilizing the 2009 CKD-*EPI* creatinine equation: 141 × min (SCr/k, 1)^α^ × max (SCr/k, 1)^-1.209^ × 0.993^Age^ [× 1.018 if female] [× 1.159 if black], where SCr represents serum creatinine (in mg/dL), k is 0.7 for females and 0.9 for males, and α is −0.329 for females and −0.411 for males. Then, according to the guidelines, the cutoff value of GFR < 60 mL/min/1.73 m^2^ and/or urinary albumin ≥ 30 mg/g was employed to determine the presence of CKD among participants with NAFLD. The samples were ultimately divided into training and validation sets in a randomized manner, adhering to a 7:3 ratio. The external validation samples were obtained from NHANES III (1988‒1994). But for the diagnosis of fatty liver, the authors referred to the research by Younossi et al., who defined moderate to severe hepatic steatosis as indicative of fatty liver disease.^19^

### Ethics statement

This cross-sectional study followed the STROBE Statement. Each participant signed a written informed consent form before their examination results were included in the NHANES database, and all data were anonymized prior to being made available to the public. The study protocol (Continuation of Protocol #2011–17; Protocol #2018–01) received approval from the NCHS Ethics Review Board. The researchers then can transform the data into a format that complies with privacy policies for subsequent analysis. In accordance with the data use guidelines of this study, the research will strictly adhere to all relevant laws and standards.

### Statistical analysis

R software (version 4.3.1, http://www.r-project.org) was employed to conduct all statistical analyses, with a significance level set at *p* < 0.05. Continuous variables were reported as median with interquartile range, the Wilcoxon rank-sum test was utilized to compare the two groups. Categorical variables were expressed as frequencies and proportions. The choice between Pearson's Chi-Square test and Fisher's exact test for intergroup comparisons was determined by the specific conditions of the analysis.

Initially, a correlation analysis was conducted on all variables to determine the presence of multicollinearity among them. Subsequently, the application of Least Absolute Shrinkage and Selection Operator (LASSO) regression became essential for identifying potential predictors within the training set, thereby reducing the impact of multicollinearity. Prior to performing LASSO regression, continuous variables were standardized, and multiclass nominal variables were transformed into dummy variables. The LASSO regression integrates the fitting of a generalized linear model with the adjustment of the model's complexity through the parameters λ. Following this, both univariate and multivariate logistic regression analyses were performed on the training dataset to assess the statistical significance of each variable's effect on the outcome. To minimize potential bias, the authors utilized variables that overlapped between the results of the LASSO regression and the multivariate logistic regression to construct a predictive nomogram, which was subsequently visualized as an online calculator. Finally, the authors evaluated the model's performance on the training set, validation set, and external validation set to confirm its predictive efficiency. Receiver Operating Characteristic (ROC) curve analysis and the Area Under the Curve (AUC) calculation were employed to assess the model's discrimination ability. Additionally, the calibration curve, derived from a bootstrap resampling method conducted 1000 times, was used to evaluate the model's prediction accuracy. The clinical utility of the nomogram model was assessed using Decision Curve Analysis (DCA).

## Results

### Characteristics of NAFLD participants

**Fig. 1:**
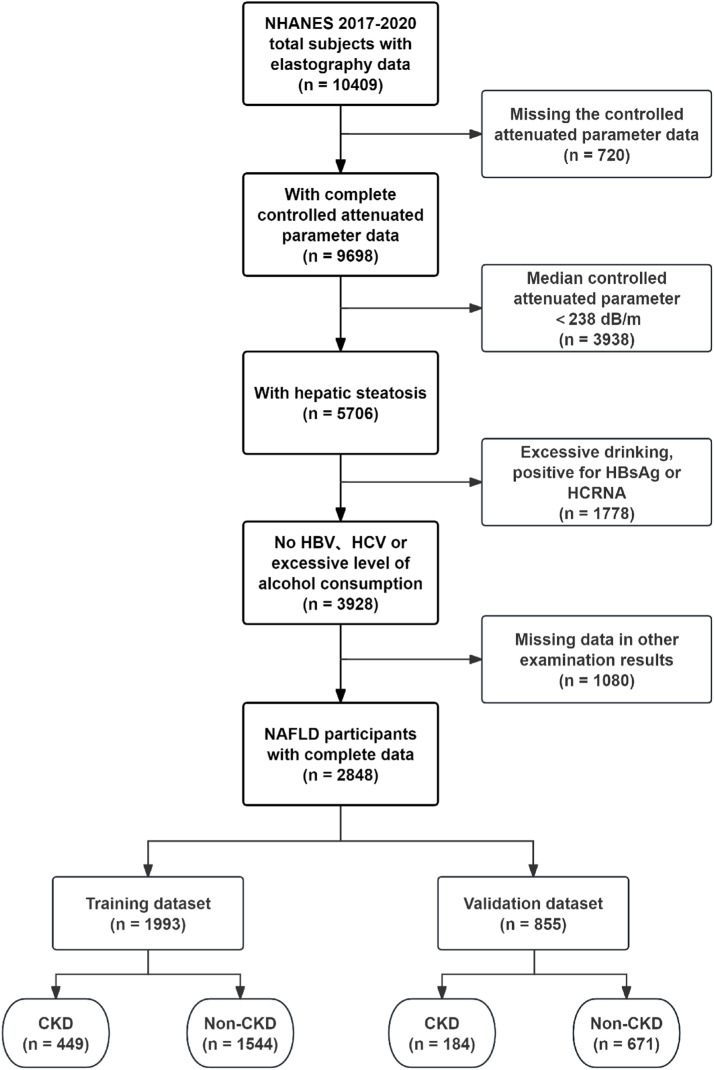
Flow chart of the study participants.

A total of 2848 participants with NAFLD were included in the study, of whom 633 had NAFLD complicated by CKD. The detailed process of data filtering is illustrated in Fig. 1. The mean age of all participants was 54.68 years, and 54.39 % were male. The prevalence of CKD among the participants was approximately 22.23 %. NAFLD participants with CKD exhibited larger demographic attributes, including age, Waist Circumference (WC), and Systolic Blood Pressure (SBP). They also had higher levels of CAP, Alkaline Phosphatase (ALP), ferritin, C-Reactive Protein (CRP), and Triglycerides (TG), as well as lower levels of Alanine Aminotransferase (ALT), albumin, High-Density Lipoprotein Cholesterol (HDL-C), and Total Cholesterol (TC) compared to those without CKD. The mean estimated GFR level in patients with concurrent CKD was 67.04 mL/min/1.73 m^2^, which was significantly lower than that of patients without CKD. Additionally, these patients exhibited a markedly higher average albumin-to-creatinine ratio of 47.37 mg/g, indicative of a more severe proteinuria condition. The basic clinical characteristics of the participants are presented in Table 1. Participants were randomly assigned in a 7:3 ratio to a training set (*n* = 1993) and a validation set (*n* = 855), with 449 participants with CKD in the training set and 184 in the validation set. There was no significant difference between the two groups in any variable (Table S1). In the external validation set, a total of 2507 participants with NAFLD were included, of whom 674 had comorbid CKD. Patients with comorbid CKD also exhibited significantly higher levels of CRP, SBP, TG, etc., compared to those without comorbid CKD (Table S2).

**Table 1:** Basic clinical characteristics of participants with NAFLD.

Variables	Non-CKD (*n* = 2215)	CKD (*n* = 633)	*P*-value
eGFR (mL/min/1.73 m^2^)	94.30 (81.02, 108.39)	67.04 (51.82, 94.26)	< 0.001
Urine albumin creatinine ratio (mg/g)	6.70 (4.51, 10.74)	47.37 (16.69, 112.22)	< 0.001
Demographic parameters			
Gender			0.836
Male	1207 (54.49 %)	342 (54.03 %)	
Female	1008 (45.51 %)	291 (45.97 %)	
Race			< 0.001
Mexican American	271 (12.23 %)	68 (10.74 %)	
Other Hispanic	232 (10.47 %)	52 (8.21 %)	
Non-Hispanic White	773 (34.90 %)	277 (43.76 %)	
Non-Hispanic Black	518 (23.39 %)	132 (20.85 %)	
Non-Hispanic Asian	330 (14.90 %)	67 (10.58 %)	
Other race	91 (4.11 %)	37 (5.85 %)	
Age (years)	54.00 (40.00, 64.00)	67.00 (56.00, 76.00)	< 0.001
BMI (kg/m^2^)	30.50 (26.90, 35.70)	31.20 (27.50, 36.00)	0.068
WC (cm)	104.10 (95.00, 116.00)	107.70 (98.50, 118.70)	< 0.001
SBP (mmHg)	123.00 (113.00, 135.00)	132.00 (118.00, 146.00)	< 0.001
DBP (mmHg)	75.00 (69.00, 82.00)	74.00 (66.00, 83.00)	0.030
Liver function parameters			
CAP (dB/m)	293.00 (266.00, 331.00)	301.00 (270.00, 338.00)	0.004
ALT (U/L)	20.00 (14.00, 28.00)	18.00 (13.00, 25.00)	< 0.001
ALP (IU/L)	75.00 (63.00, 91.00)	80.00 (66.00, 98.00)	< 0.001
AST (U/L)	19.00 (16.00, 24.00)	19.00 (15.00, 24.00)	0.175
GGT (IU/L)	23.00 (16.00, 33.00)	23.00 (17.00, 34.00)	0.251
TB (mg/dL)	0.40 (0.30, 0.50)	0.40 (0.30, 0.60)	0.561
TP (g/L)	72.00 (69.00, 74.00)	72.00 (69.00, 75.00)	0.934
Serum biochemical parameters			
Albumin (g/L)	41.00 (39.00, 43.00)	40.00 (38.00, 42.00)	< 0.001
Iron (μmol/L)	14.70 (11.30, 18.50)	14.10 (11.10, 17.70)	0.030
Ferritin (μg/L)	119.00 (59.80, 212.50)	131.00 (67.20, 239.00)	0.002
TIBC (μmol/L)	57.31 (52.48, 62.69)	56.60 (50.69, 62.51)	0.021
TSAT (%)	26.00 (20.00, 33.00)	25.00 (19.00, 32.00)	0.194
CRP (mg/L)	2.23 (1.03, 4.71)	2.64 (1.16, 5.91)	< 0.001
HDL-C (mmol/L)	1.24 (1.03, 1.47)	1.19 (1.01, 1.45)	0.006
TC (mmol/L)	4.81 (4.16, 5.51)	4.55 (3.90, 5.35)	< 0.001
TG (mg/dL)	126.00 (90.50, 178.00)	143.00 (100.00, 209.00)	< 0.001

NAFLD, nonalcoholic fatty liver disease; CKD, chronic kidney disease; eGFR, estimated glomerular filtration rate; BMI, body mass index; WC, waist circumference; SBP, systolic blood pressure; DBP, diastolic blood pressure; CAP, controlled attenuated parameter; ALT, alanine aminotransferase; ALP, alkaline phosphatase; AST, aspartate aminotransferase; GGT, γ-glutamyl transpeptidase; TB, total bilirubin; TP, total protein; TIBC, total iron binding capacity; TSAT transferrin saturation; CRP, C-reactive protein; HDL-C, high-density lipoprotein cholesterol; TC, total cholesterol; TG, triglycerides.

### Identification of significant variables

**Fig. 2:**
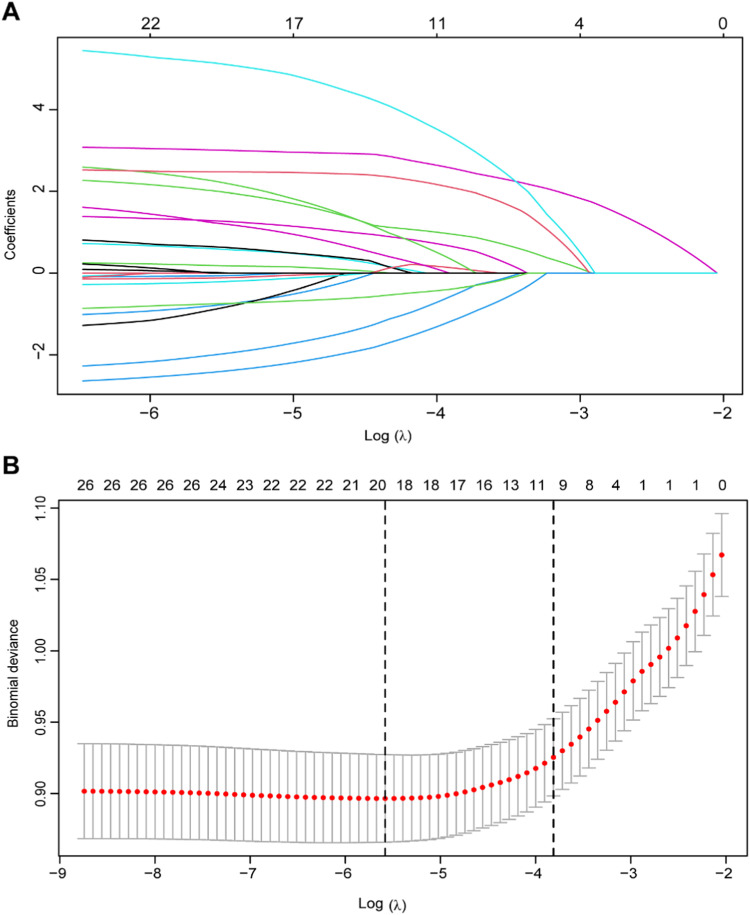
Clinical feature selection using the LASSO logistic regression model. (A) The trajectory of the coefficients of potential predictors as log (λ) increases. (B) 10-fold cross-validation results for the penalty parameter.

The correlation degree of each variable was presented in the form of a heat map, as illustrated in Figure S1. The heat map revealed the presence of multicollinearity among the majority of variables, necessitating the use of appropriate regression methods for variable selection. Consequently, LASSO regression was employed to identify the predictive variables within the training set. Fig. 2A depicts the changes in the regression coefficients of each variable across different values of λ. The trajectory of each variable’s coefficient is represented by distinct curves in various colors. As the logarithm of λ increases, the regression coefficients converge, resulting in a decrease in the number of independent variables included in the model. The results of 10-fold cross-validation are shown in Fig. 2B. The vertical dotted line on the left indicates the λ value with the smallest deviation and its corresponding number of variables (λ.min), while the dotted line on the right represents one standard error of λ.min (λ.1se). In this study, the optimal value was determined to be λ.1se, leading to the selection of ten variables with non-zero coefficients, including age, WC, SBP, ALP, TP, albumin, CRP, HDL-C, TC, and TG. Univariate and multivariate logistic regression analyses were also conducted on the training set, revealing that seven variables ‒ age, SBP, albumin, ferritin, CRP, TC, and TG ‒ were independently associated with CKD (Table 2).

**Table 2:** Univariate and multivariate logistic regression analysis of CKD risk predictors in the training dataset.

Variables	Univariate			Multivariate		
	OR	95 % CI	*P*-value	OR	95 % CI	*P*-value
Demographic parameters						
Gender						
Male			Ref.			
Female	0.996	0.807–1.230	0.973			
Race						
Mexican American			Ref.			
Other Hispanic	0.924	0.578–1.471	0.740			
Non-Hispanic White	1.426	1.004–2.055	0.052			
Non-Hispanic Black	1.011	0.688–1.500	0.956			
Non-Hispanic Asian	0.758	0.483–1.186	0.225			
Other race	1.453	0.808–2.565	0.203			
Age (years)	1.056	1.048–1.065	< 0.001	1.053	1.041–1.066	< 0.001
BMI (kg/m^2^)	1.013	0.999–1.027	0.072			
WC (cm)	1.013	1.007–1.020	< 0.001			
SBP (mmHg)	1.025	1.020–1.031	< 0.001	1.017	1.009–1.026	< 0.001
DBP (mmHg)	0.993	0.984–1.003	0.176			
Liver function parameters						
CAP (dB/m)	1.004	1.001–1.006	0.001			
ALT (U/L)	0.988	0.979–0.996	0.004			
ALP (IU/L)	1.010	1.006–1.014	< 0.001			
AST (U/L)	0.994	0.981–1.005	0.308			
GGT (IU/L)	1.002	0.998–1.005	0.283			
TB (mg/dL)	1.046	0.712–1.512	0.813			
TP (g/L)	1.006	0.982–1.031	0.613			
Serum biochemical parameters						
Albumin (g/L)	0.917	0.888–0.948	< 0.001	0.919	0.875–0.964	< 0.001
Iron (μmol/L)	0.976	0.957–0.995	0.012			
Ferritin (μg/L)	1.001	1.000–1.002	< 0.001	1.001	1.000–1.002	0.030
TIBC (μmol/L)	0.987	0.975–1.000	0.046			
TSAT (%)	0.993	0.983–1.003	0.191			
CRP (mg/L)	1.032	1.019–1.046	< 0.001	1.020	1.006–1.035	0.008
HDL-C (mmol/L)	0.620	0.449–0.848	0.003			
TC (mmol/L)	0.817	0.736–0.905	< 0.001	0.730	0.639–0.832	< 0.001
TG (mg/dL)	1.003	1.002–1.004	< 0.001	1.004	1.002–1.005	< 0.001

CKD, chronic kidney disease; BMI, body mass index; WC, waist circumference; SBP, systolic blood pressure; DBP, diastolic blood pressure; CAP, controlled attenuated parameter; ALT, alanine aminotransferase; ALP, alkaline phosphatase; AST, aspartate aminotransferase; GGT, γ-glutamyl transpeptidase; TB, total bilirubin; TP, total protein; TIBC, total iron binding capacity; TSAT transferrin saturation; CRP, C-reactive protein; HDL-C, high-density lipoprotein cholesterol; TC, total cholesterol; TG, triglycerides; OR, odds ratio; CI, confidence interval; Ref, reference.

### Development and assessment of the predictive nomogram

**Fig. 3:**
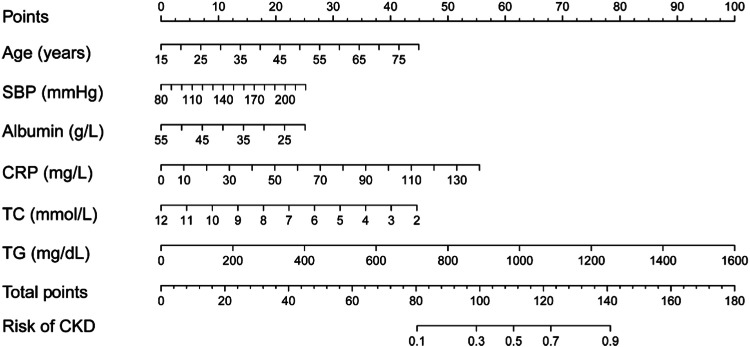
Nomogram for predicting the risk of CKD in adults with NAFLD.

**Fig. 4:**
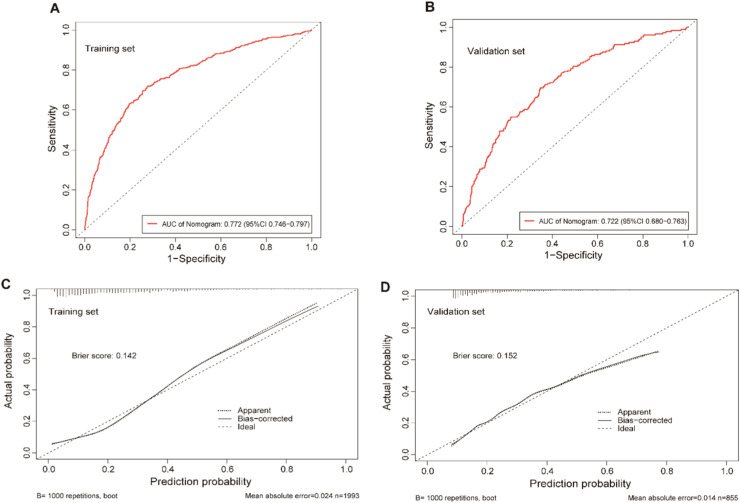
The receiver operating characteristic (ROC) curves and calibration curves of the nomogram model. (A) ROC curves of the nomogram in the training set, the AUC was 0.772 (95 % CI 0.746–0.797). (B) ROC curve of the nomogram in the validation group, the AUC was 0.722 (95 % CI 0.680–0.763). (C) Calibration curve for the training set, the Brier score was 0.142. (D) Calibration curve for the validation set, the Brier score was 0.152. The x-axis represents the predicted probability, while the y-axis represents the actual probability in the calibration curves.

The clinical predictive variables were selected based on the overlap between the results of LASSO regression and multivariate logistic regression (Fig. S2). Ultimately, six independent predictors were utilized to establish a prediction model, which is presented in the form of a nomogram. These predictors include age, SBP, serum albumin, CRP, TC, and TG. Distinct values for each variable correspond to specific scores, which can be aggregated to determine the total score for each individual, locate this total score on the “Total Points” axis and draw a vertical line downward until it intersects with the “Risk of CKD” axis. The value obtained at this intersection represents the probability of CKD comorbidity (Fig. 3). In order to enhance the accessibility of the nomogram for researchers, the authors have developed a web-based dynamic version which can be directly accessed on the Internet through https://gdmudz.shinyapps.io/dynnomapp/. The discriminative ability of the prediction model was evaluated using ROC curve analysis. The AUC of the training set was 0.772, with a 95 % Confidence Interval (95 % CI) of 0.746 to 0.797 (Fig. 4A). In contrast, the AUC for the validation set was 0.722 with a 95 % CI of 0.680 to 0.763 (Fig. 4B). The calibration curves generated using the bootstrap resampling method reveal that the Brier scores in the training set (Fig. 4C) and validation set (Fig. 4D) were 0.142 and 0.152, respectively, indicating a strong concordance between the actual and predicted probabilities. The DCA indicated that the model provided a net benefit within the risk threshold range of 0.1 to 0.7 for the training set and 0.1 to 0.5 for the validation set (Fig. S3). The AUC for the external validation set was 0.748, with a 95 % CI of 0.728 to 0.769, and the Brier score was 0.182 (Fig. S4).

## Discussion

This study was conducted on a population with NAFLD to investigate the likelihood of comorbidity between NAFLD and CKD, utilizing commonly employed detection indicators in NAFLD screening, rather than those specific to CKD, as potential predictors. The prediction model, presented in the form of a nomogram, provides a highly convenient method for obtaining calculation results. The evaluation results indicate that the model the authors established demonstrates good accuracy and clinical utility, allowing for a preliminary estimation of CKD comorbidity in the NAFLD population. In the DCA results, the net benefits for the training set and validation set were observed within the risk threshold ranges of 0.1 to 0.7 and 0.1 to 0.5, respectively. According to the present study’s model, 88 patients in the training set and 35 patients in the validation set fell within their respective risk threshold ranges. This suggests that if intervention decisions are guided by the model's computational outcomes, approximately one-quarter of patients with comorbidities would fall within the range of positive benefits. The model's performance in external validation further indicates its excellent prediction accuracy and generalization ability. Furthermore, the findings of this study may provide insights for future research into the pathological mechanisms underlying the comorbidity of NAFLD and CKD.

In fact, as early as 2008, multiple studies have demonstrated a significant association between NAFLD and an elevated risk of CKD,^20^^,^^21^ which initiated a series of subsequent epidemiological investigations and meta-analyses.^22^ The presence of NAFLD was found to be significantly associated with approximately a twofold increase in both the prevalence and incidence of CKD. These associations remained significant even after adjusting for confounding factors such as diabetes status and traditional risk factors for CKD.^23^ The potential mechanisms linking NAFLD to CKD may stem from the expansion and inflammation of visceral adipose tissue, as well as other pathophysiological pathways not solely associated with liver inflammation. One such pathway involves plasma adiponectin, a polypeptide secreted by adipocytes that possesses antidiabetic and anti-inflammatory properties.^24^ Dilated and inflamed visceral adipose tissue releases a variety of molecules, including interleukin-6, tumor necrosis factor-alpha, and other pro-inflammatory cytokines, which may contribute to the pathogenesis of insulin resistance and kidney injury.^24^^,^^25^ Plasma adiponectin levels exhibit an inverse correlation with the histological severity of NAFLD.^26^ Reduced adiponectin levels suppress the activation of 5′-AMP-activated protein kinase, a crucial molecule that regulates energy metabolism, ultimately leading to organ damage, including liver cirrhosis and kidney disease. In addition to mechanisms related to insulin resistance and lipid metabolism, Lonardo et al. also identified genetic polymorphisms, musculoskeletal diseases, and a pathological hepatorenal reflex as potential factors contributing to the development of CKD in individuals with NAFLD. They speculated that the presence of the rs738409 *C* > *G* p.I148M variant in the PNPLA3 gene, which is predominantly expressed in the liver, may activate pericytes, resulting in the development of renal steatosis and fibrosis.^27^

Although the precise pathological mechanism linking NAFLD and CKD remains elusive, it is well-established that NAFLD is associated with an increased risk of CKD comorbidity. So the authors have used multiple data processing methods to identify key indicators in NAFLD patients for concurrent CKD estimation; both LASSO regression and logistic regression were employed for variable screening. Ultimately, six common indicators for detecting NAFLD were selected as variables to assess the likelihood of NAFLD complicated by CKD: age, SBP, albumin, CRP, TC, TG. The number of variables retained by LASSO regression exceeded that of logistic regression may be due to their differing methodologies. This discrepancy can vary across datasets because LASSO does not consistently reduce all unimportant variable coefficients to zero, particularly when variables are highly correlated. Instead, it may arbitrarily select one variable from a set of correlated predictors and retain its coefficient, but it can effectively address multicollinearity issues. In contrast, logistic regression lacks an inherent variable selection mechanism; it estimates the regression coefficients for all provided variables and requires the manual establishment of a threshold for eliminating variables or combining them with other selection techniques. Therefore, integrating these two approaches for variable screening can help minimize potential biases in the analysis.

The indicators the authors have identified have been extensively examined in prior studies. CRP, in particular, is a nonspecific biomarker of the systemic inflammatory response during the acute phase, primarily synthesized by hepatocytes, although it may also be expressed in other cells, including renal tubular cells.^28^^,^^29^ A cross-sectional study of adults in Taiwan indicates that CRP may serve as a useful surrogate marker for assessing the risk of CKD.^30^ Aging is also linked to declines in various renal functions, including the glomerular filtration rate.^31^ These calculations support this observation, indicating that older age correlates with a higher probability of NAFLD complicated by CKD. Systolic hypertension is common among individuals with NAFLD, particularly among the elderly, and is a significant risk factor for cardiovascular disease, heart failure, and CKD. Effective management of SBP has been associated with a substantial reduction in the incidence of these complications. Notably, SBP tends to increase as CKD progresses, even after adjusting for age and gender.^32^ Both NAFLD and CKD patients exhibit varying degrees of dyslipidemia; thus, two lipid parameters, TC and TG, were identified as predictor variables. The mean serum TG level in patients with CKD increases almost linearly, beginning in the early stages and peaking during stages IV and V.^33^ However, current studies on the association between TC and CKD are limited, although some align with the present findings. For instance, Weiner et al. discovered a significant association between lower TC levels and increased all-cause mortality in hemodialysis patients. Additionally, they observed a close correlation between elevated CRP levels, reduced serum albumin levels, and hypocholesterolemia.^34^ It can be seen that among the potential mechanisms linking NAFLD and CKD, CRP may serve as a pivotal factor.

Despite the strong predictive performance demonstrated by the established model, this study has several limitations. First, the present research employed a cross-sectional design, which cannot establish a causal relationship between NAFLD and CKD. Second, the sample size in this study is relatively small, which may introduce bias into the analysis results. Third, the relationship between certain variables and CKD remains unclear, with conflicting findings reported in different studies.

## Conclusion

This study established and validated a nomogram model to assess the probability of CKD in U.S. adults with NAFLD, utilizing six routine NAFLD detection indicators: age, SBP, serum albumin, CRP, TC, and TG. The validation results demonstrate that the model exhibits strong predictive performance. In practice, CKD often presents noticeable symptoms primarily during the middle and late stages; however, implementing appropriate nutritional and exercise interventions can effectively slow the progression of CKD. Therefore, this model will enable researchers and clinicians to quickly make a preliminary estimation of the likelihood of comorbid CKD in patients with NAFLD based on their fundamental information. Nevertheless, more prospective and rigorous studies are needed to further investigate the mechanisms underlying the complications of NAFLD and CKD in the future.

## Funding

This study was funded by the BYHEALTH Nutrition and Health Research Foundation (TY202101001), the 10.13039/501100001809National Natural Science Foundation of China (82,273,622, 82,203,361), and the 10.13039/501100003785Guangdong Medical Research Foundation (A2023341).

## CRediT authorship contribution statement

**Dazhang Deng:** Investigation, Data curation, Writing – original draft. **Yutong Xie:** Methodology, Investigation, Validation, Writing – review & editing. **Ya Wang:** Investigation, Visualization. **Wanhan Song:** Conceptualization, Methodology, Writing – review & editing. **Yuguo Liu:** Investigation, Data curation. **Bin Liu:** Funding acquisition, Writing – review & editing. **Honghui Guo:** Resources, Project administration, Funding acquisition, Supervision.

## Declaration of competing interest

The authors declare that they have no known competing financial interests or personal relationships that could have appeared to influence the work reported in this paper.

## Appendix Supplementary materials

Image, application 1
