# Supplementary material for: Construction and validation of a nomogram for detecting chronic kidney disease in patients with nonalcoholic fatty liver disease: Insights from the NHANES database
Source: Clinics (Sao Paulo). 2025 May 7;80:100686. doi: 10.1016/j.clinsp.2025.100686 (PMC12133694; doi:10.1016/j.clinsp.2025.100686)

**CLINICS-D-24-00842_Supplementary Materials**

**Supplementary Materials**

Supplementary material associated with this article can be found in the online version.

**Supplementary Table S1** Characteristics of the study participants between training set and validation set

| **Variables** | **Training set**  **(n = 1993)** | **Validation set**  **(n = 855)** | **p-value** |
| --- | --- | --- | --- |
| CKD (%) | 449.00 (22.53%) | 184.00 (21.52%) | 0.553 |
| Demographic parameters |  |  |  |
| Gender |  |  | 0.017 |
| Male | 1,055 (52.94%) | 494 (57.78%) |  |
| Female | 938 (47.06%) | 361 (42.22%) |  |
| Race |  |  | 0.355 |
| Mexican American | 233 (11.69%) | 106 (12.40%) |  |
| Other Hispanic | 212 (10.64%) | 72 (8.42%) |  |
| Non-Hispanic White | 722 (36.23%) | 328 (38.36%) |  |
| Non-Hispanic Black | 462 (23.18%) | 188 (21.99%) |  |
| Non-Hispanic Asian | 280 (14.05%) | 117 (13.68%) |  |
| Other race | 84 (4.21%) | 44 (5.15%) |  |
| Age (years) | 57.00 (42.00, 68.00) | 57.00 (44.00, 68.00) | 0.888 |
| BMI (kg/m^2^) | 30.60 (26.90, 35.80) | 30.80 (27.20, 35.40) | 0.795 |
| WC (cm) | 104.90 (95.50, 117.00) | 105.60 (96.20, 116.30) | 0.758 |
| SBP (mmHg) | 124.00 (113.00, 138.00) | 125.00 (114.50, 138.00) | 0.483 |
| DBP (mmHg) | 75.00 (68.00, 82.00) | 75.00 (68.00, 82.00) | 0.862 |
| Liver function parameters |  |  |  |
| CAP (dB/m) | 294.00 (266.00, 331.00) | 298.00 (268.50, 334.50) | 0.114 |
| ALT (U/L) | 19.00 (14.00, 27.00) | 19.00 (15.00, 28.00) | 0.038 |
| ALP (IU/L) | 76.00 (63.00, 93.00) | 76.00 (65.00, 91.00) | 0.772 |
| AST (U/L) | 19.00 (16.00, 24.00) | 20.00 (16.00, 24.00) | 0.064 |
| GGT (IU/L) | 22.00 (16.00, 32.00) | 23.00 (17.00, 33.00) | 0.026 |
| TB (mg/dL) | 0.40 (0.30, 0.60) | 0.40 (0.30, 0.50) | 0.500 |
| TP (g/L) | 72.00 (69.00, 74.00) | 72.00 (69.00, 74.00) | 0.243 |
| Serum biochemical parameters |  |  |  |
| Albumin (g/L) | 41.00 (39.00, 43.00) | 41.00 (39.00, 43.00) | 0.176 |
| Iron (μmoL/L) | 14.50 (11.30, 18.30) | 14.90 (11.10, 18.40) | 0.649 |
| Ferritin (μg/L) | 121.00 (61.70, 216.00) | 122.00 (61.10, 219.00) | 0.598 |
| TIBC (μmoL/L) | 56.95 (51.94, 62.33) | 57.85 (52.66, 63.58) | 0.034 |
| TSAT (%) | 26.00 (20.00, 32.00) | 26.00 (19.00, 32.00) | 0.711 |
| CRP (mg/L) | 2.34 (1.05, 4.96) | 2.27 (1.06, 4.86) | 0.682 |
| HDL-C (mmoL/L) | 1.24 (1.03, 1.47) | 1.19 (1.03, 1.42) | 0.018 |
| TC (mmoL/L) | 4.76 (4.11, 5.51) | 4.68 (4.06, 5.43) | 0.127 |
| TG (mg/dL) | 129.00 (92.00, 182.00) | 133.00 (93.00, 189.50) | 0.211 |

CKD, Chronic Kidney Disease; BMI, Body Mass Index; WC, Waist Circumference; SBP, Systolic Blood Pressure; DBP, Diastolic Blood Pressure; CAP, Controlled Attenuated Parameter; ALT, Alanine Aminotransferase; ALP, Alkaline Phosphatase; AST, Aspartate Aminotransferase; GGT, γ-Glutamyl Transpeptidase; TB, Total Bilirubin; TP, Total Protein; TIBC, Total Iron Binding Capacity; TSAT, Transferrin Saturation; CRP, C-Reactive Protein; HDL-C, High-Density Lipoprotein Cholesterol; TC, Total Cholesterol; TG, Triglycerides.

**Supplementary Table S2** Characteristics of the NAFLD participants in external validation set.

| **Variables** | **Non-CKD (n = 1833)** | **CKD (n = 674)** | **p-value** |
| --- | --- | --- | --- |
| Gender |  |  | <0.001 |
| Male | 938 (51.17%) | 287 (42.58%) |  |
| Female | 895 (48.83%) | 387 (57.42%) |  |
| Race |  |  | 0.004 |
| White | 1332 (72.67%) | 510 (75.67%) |  |
| Black | 404 (22.04%) | 146 (21.66%) |  |
| Mexican American | 0 (0.00%) | 1 (0.15%) |  |
| Other race | 97 (5.29%) | 17 (2.52%) |  |
| Age (years) | 43.00 (33.00, 57.00) | 63.00 (51.00, 69.00) | <0.001 |
| SBP (mmHg) | 121.00 (111.00, 133.00) | 135.00 (122.00, 151.00) | <0.001 |
| Albumin (g/L) | 41.00 (39.00, 44.00) | 41.00 (39.00, 43.00) | 0.018 |
| CRP (mg/L) | 2.10 (2.10, 6.00) | 3.30 (2.10, 8.00) | <0.001 |
| TC (mmoL/L) | 5.30 (4.63, 6.05) | 5.72 (5.02, 6.47) | <0.001 |
| TG (mg/dL) | 150.00 (99.00, 225.00) | 179.50 (128.00, 268.75) | <0.001 |
| eGFR (mL/min/1.73 m^2^) | 80.08 (70.91, 91.57) | 57.84 (51.55, 72.62) | <0.001 |
| Urine albumin creatinine ratio (mg/g) | 5.56 (3.14, 9.47) | 31.47 (7.04, 81.77) | <0.001 |

CKD, Chronic Kidney Disease; SBP, Systolic Blood Pressure; CRP, C-Reactive Protein; TC, Total Cholesterol; TG, Triglycerides; eGFR, estimated Glomerular Filtration Rate.

**Supplementary Figure S1** Heat map of the candidate variables. The size and color of the circle, as well as the color of the grid, serve as indicators for quantifying the degree of correlation between the variables. Blue represents a positive correlation, while brown indicates a negative correlation. The darker the color, the stronger the correlation. The significance of the correlation analysis is indicated by symbols, *p < 0.05, **p < 0.01 and ***p < 0.001.

**
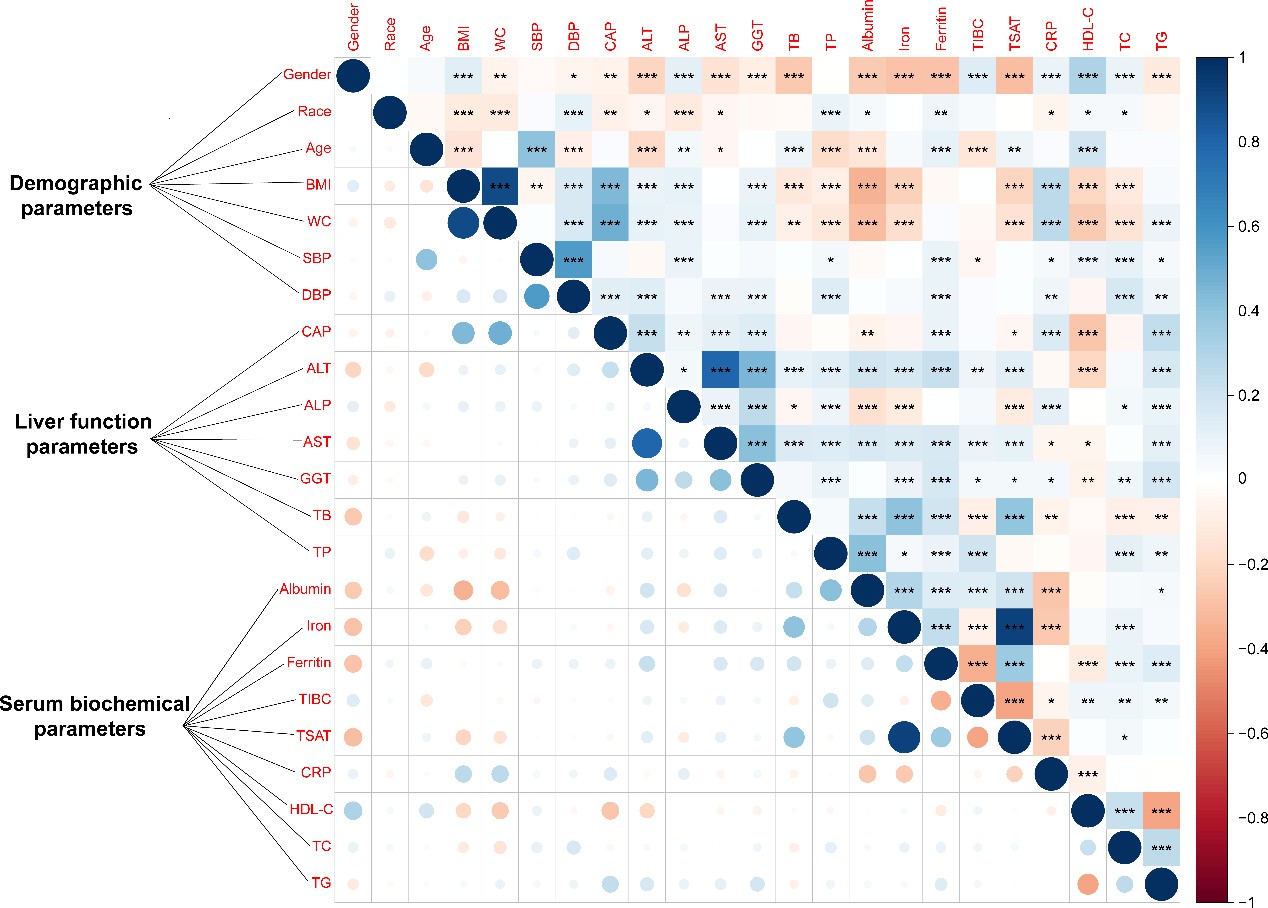
**

**Supplementary Figure S2** The overlapping predictor variables identified by LASSO regression and multivariate logistic regression. LASSO regression screened out ten variables, logistic regression screened out seven variables, and there were six variables that overlapped between the two methods.


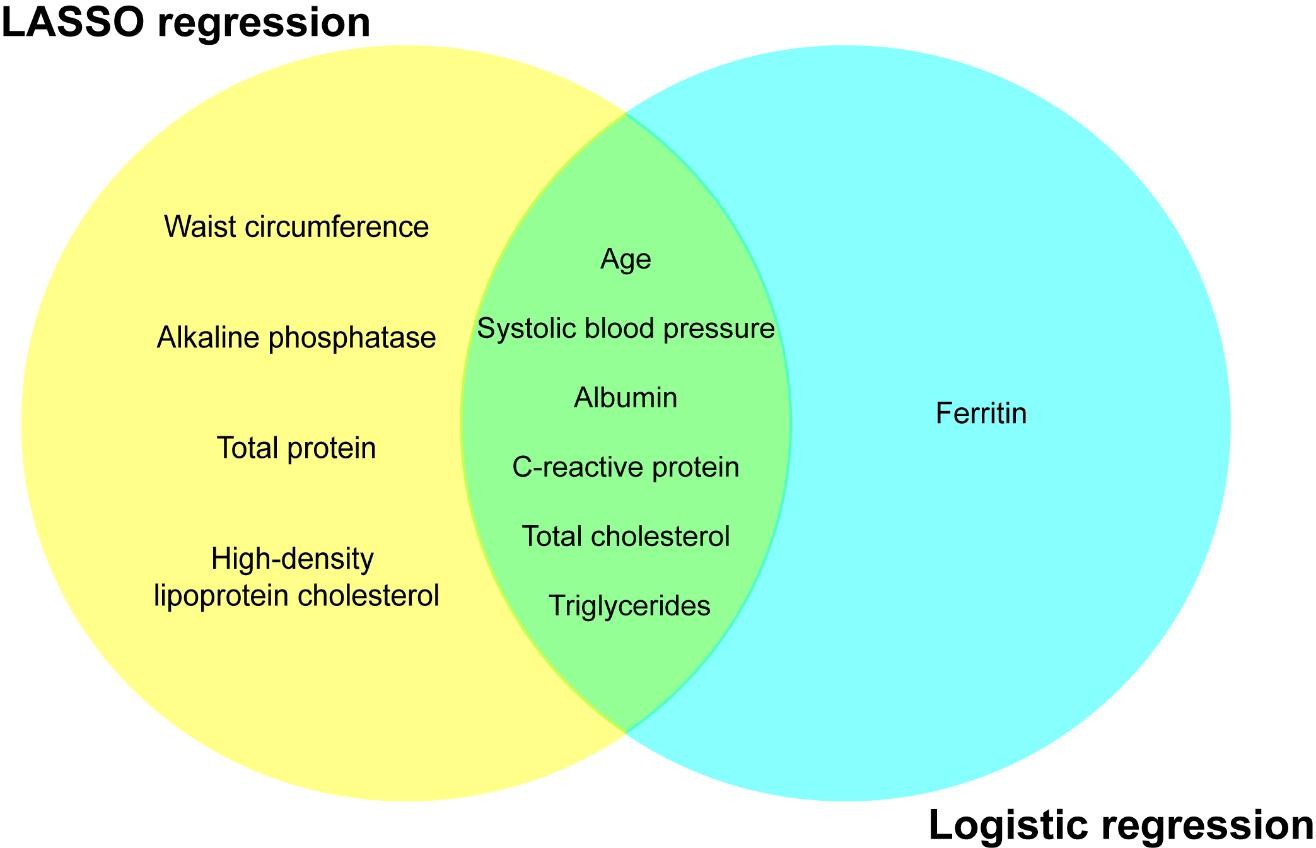


**Supplementary Figure S3** Decision Curve Analysis (DCA) of the nomogram. (A) DCA of the nomogram in the training set. (B) DCA of the nomogram in the validation set. The blue line represents clinical intervention for all objects, while the horizontal black line signifies no intervention for any objects, and the red line denotes the benefit of intervention based on the predicted results of the nomogram.


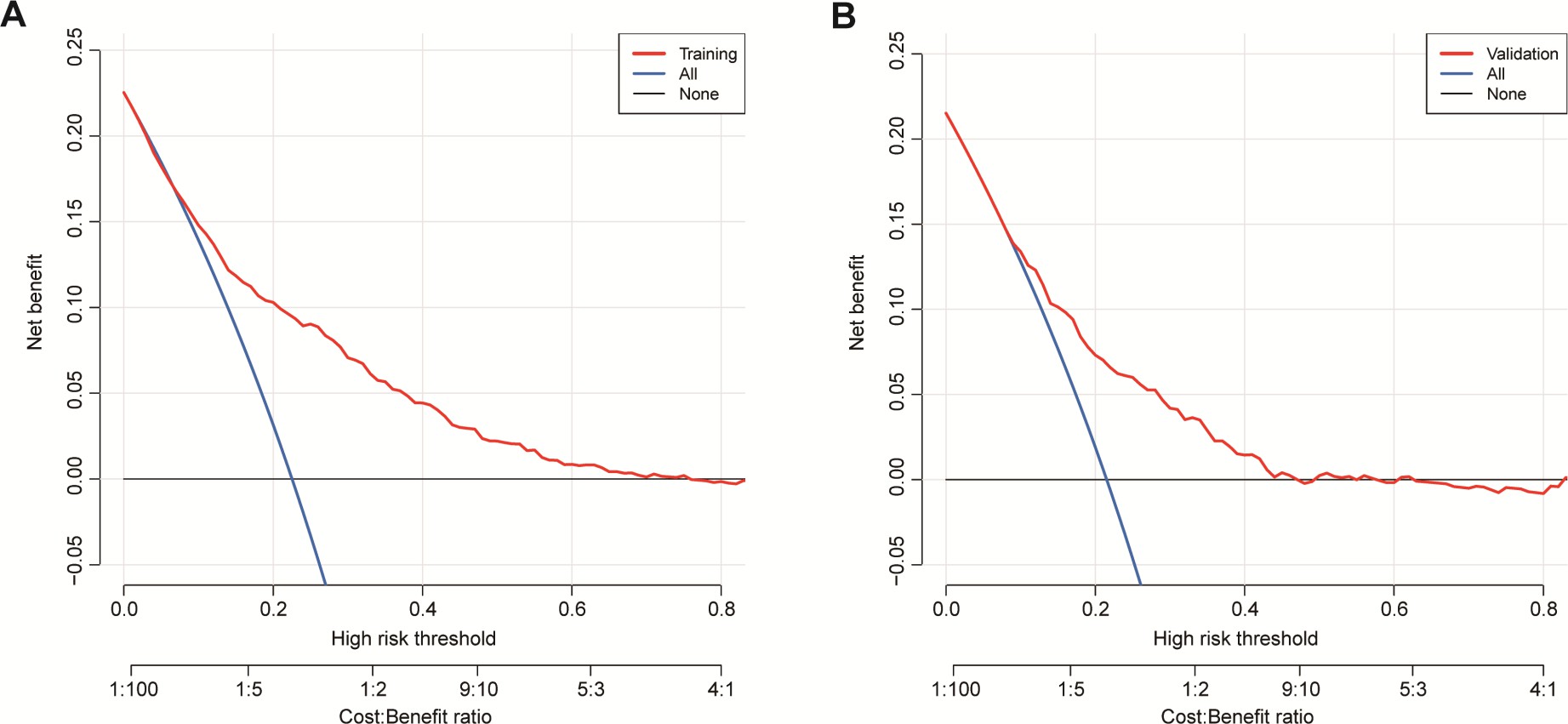


**Supplementary Figure S4** External validation of the nomogram. (A) ROC curves of the nomogram in the external validation set, the AUC was 0.748 (95% CI 0.728–0.769). (B) Calibration curve for the external validation set, the Brier score was 0.182. (C) DCA of the nomogram in the external validation set.


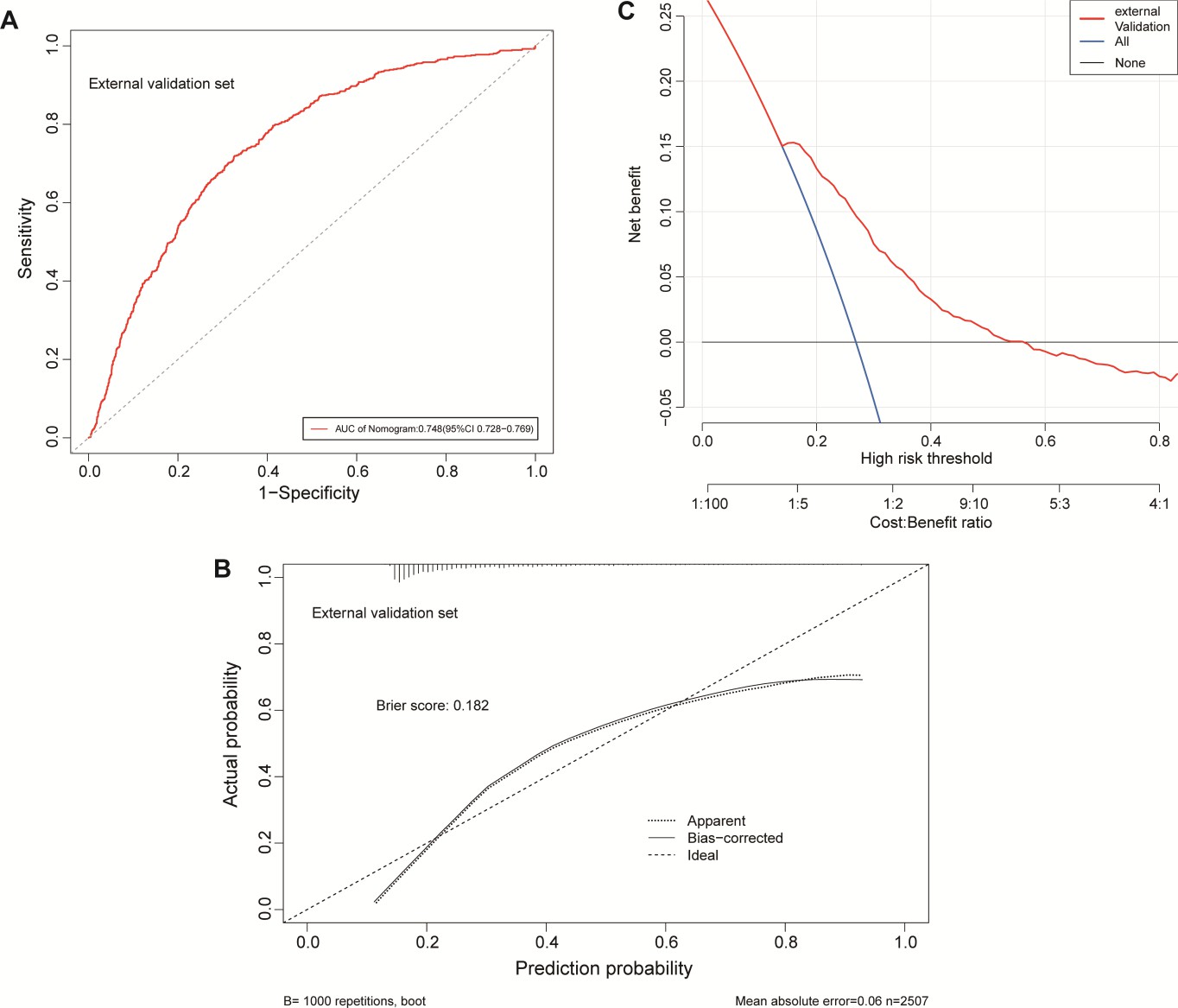

Supplement: Supplementary file 1 [file mmc1.docx]
